# Emerging perspectives on precision therapy for Parkinson’s disease: multidimensional evidence leading to a new breakthrough in personalized medicine

**DOI:** 10.3389/fnagi.2024.1417515

**Published:** 2024-07-04

**Authors:** Qiaoli Wang, Xuan Gu, Le Yang, Yan Jiang, Jiao Zhang, Jinting He

**Affiliations:** ^1^Department of Neurology, China-Japan Union Hospital of Jilin University, Changchun, China; ^2^Department of Trauma center, China-Japan Union Hospital of Jilin University, Changchun, China; ^3^Department of Endocrinology, The People’s Hospital of Jilin Province, Changchun, China

**Keywords:** Parkinson’s disease, allele-specific targeting, A-synuclein_3_, gene variant, precision therapy

## Abstract

PD is a prevalent and progressive neurodegenerative disorder characterized by both motor and non-motor symptoms. Genes play a significant role in the onset and progression of the disease. While the complexity and pleiotropy of gene expression networks have posed challenges for gene-targeted therapies, numerous pathways of gene variant expression show promise as therapeutic targets in preclinical studies, with some already in clinical trials. With the recognition of the numerous genes and complex pathways that can influence PD, it may be possible to take a novel approach to choose a treatment for the condition. This approach would be based on the symptoms, genomics, and underlying mechanisms of the disease. We discuss the utilization of emerging genetic and pathological knowledge of PD patients to categorize the disease into subgroups. Our long-term objective is to generate new insights for the therapeutic approach to the disease, aiming to delay and treat it more effectively, and ultimately reduce the burden on individuals and society.

## 1 Introduction

With the accelerated aging of society, neurological disorders are increasingly becoming the leading cause of disability worldwide, with the incidence of Parkinson’s disease (PD) rising at an even faster rate (Collaborators, 2019; Armstrong and Okun, 2020). In 2016, an estimated 6.1 million persons were diagnosed with PD globally, which is 2.4 times the number of diagnoses in 1990 (Armstrong and Okun, 2020). It is estimated that approximately 1,238,000 people will be living with a PD diagnosis in the United States in 2030 (Marras et al., 2018). The disease presents with a wide range of clinical manifestations, including motor symptoms like resting tremor, rigidity, bradykinesia, and postural balance disorders, as well as non-motor symptoms such as rapid eye movement sleep behavior disorder (RBD), depression, autonomic dysfunction, cognitive deficits, orthostatic hypotension, and pain (Emamzadeh and Surguchov, 2018). These symptoms not only diminish the quality of life for patients but also impose a burden on their families and society. Among the broad descriptions of PD, the emergence and development of motor and non-motor symptoms may vary significantly among individuals (Berg et al., 2021).

Although lewy body disorders and dopamine depletion are thought to play a major part in the pathogenesis of PD, abnormal aggregation of -synuclein, mitochondrial functional disorders, disturbances in immune homeostasis, and lysosomal dysfunction are also thought to play significant roles as well (Greenland et al., 2019; Berg et al., 2021). It is imperative to acknowledge the heterogeneity of PD among individuals, as evidenced by variations in anatomical involvement, clinical severity, and diverse pathological changes. Furthermore, multiple cellular, organ, and systematic procedures, along with risk factors, could have a significance in the causation and spatial progression of Parkinson’s disease (Doppler et al., 2017; Knudsen et al., 2018; Johnson et al., 2019; Berg et al., 2021).

PD exhibits significant diversity in motor and non-motor symptoms, biomarkers, age of onset, etiological factors, and causal genes. This heterogeneity challenges the perception of PD as a singular entity, emphasizing its classification as a syndrome with a spectrum of overlapping clinical and pathological subtypes (Titova et al., 2017; Berg et al., 2021). Through analysis of patient subtypes, including detailed differentiation based on onset, clinical presentation and understanding of underlying disease mechanisms, is imperative for the development of personalized therapeutic interventions (Marras and Lang, 2013; Fereshtehnejad et al., 2017).

**Figure 1:**
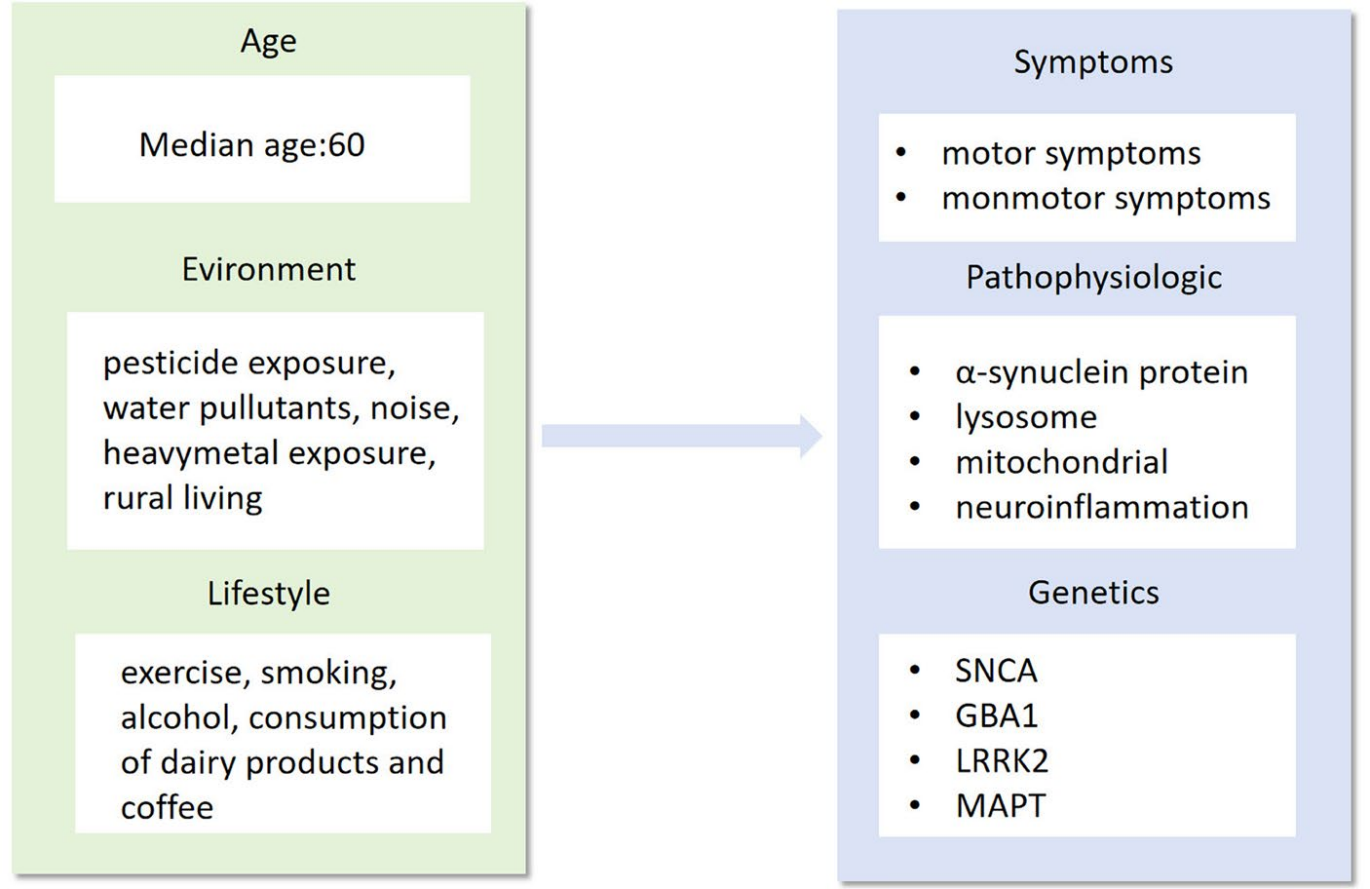
Age, environment and lifestyle are the main factors that influence the phenotype, pathogenesis and genotype of Parkinson’s disease. As a highly complex neurological syndrome, Parkinson’s disease needs to be researched in all aspects of its precise treatment, from influencing factors to disease phenotypes.

**Figure 2:**
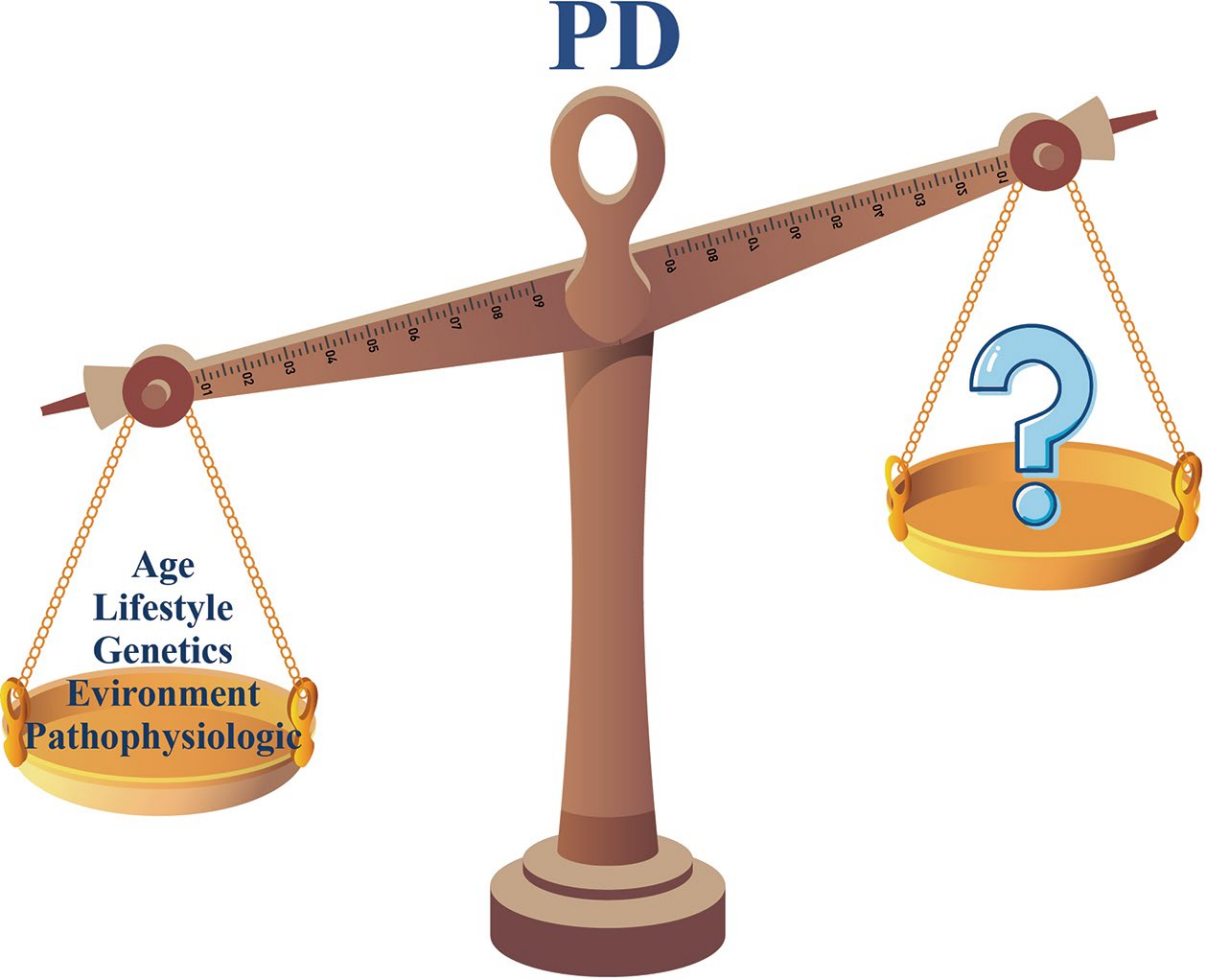
How do we balance the scales?

Currently, there is no conclusive evidence to support the effectiveness of treatments that can alter the course of PD. Existing therapeutic strategies primarily focus on managing symptoms, a reactive approach that inevitably leads to increasing disability and a diminishing sense of independence as the disease progresses. The complex medical, social, and economic challenges posed by Parkinson’s underscore the urgent need for interventions that can modify its progression and enhance the quality of life of patients. Given the intricate genetic underpinnings of Parkinson’s, a deeper understanding of its functional genomics is revealing shared disease mechanisms. This knowledge holds immense potential to significantly reshape clinical diagnostic and management approaches, paving the way for the development of therapies that can modify the course of the disease and improve patient outcomes (Ye et al., 2023). In this review, we will discuss clinical evidence from genetic, pathological, immunological, and epidemiological studies concerning animal studies. These studies have helped to explore and validate targets that may serve as therapeutic interventions in the disease, to alleviate or treat PD (Figures 1, 2).

## 2 Clinical heterogeneity in PD

**Figure 3:**
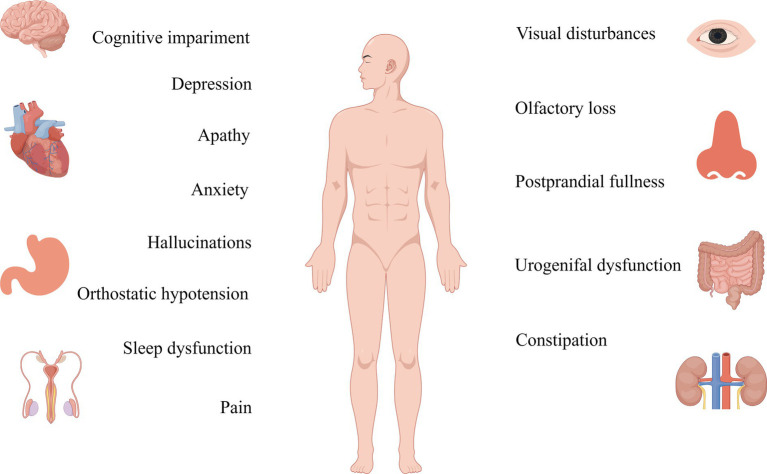
Non-motor symptoms of Parkinson’s are detected more than a decade before the onset of motor symptoms, and their therapeutic targets should be studied in greater depth.

Although clinical diagnostic criteria for PD have been revised, its diagnosis remains challenging due to the variety of clinical features and lack of specific biomarkers. The current clinical diagnosis of PD relies primarily on history and physical examination (Armstrong and Okun, 2020). The complexity and variety of clinical signs mark the start of a personalized approach to PD (Table 1; Figure 3). Before delving into precision medicine, it is helpful to examine what is currently understood about the heterogeneity of clinical PD.

**Table 1:** Clinical manifestations of motor and non-motor symptoms in Parkinson's disease.

Symptom		Key elements
Motor symptoms		
Resting tremor		69% patients (Hughes et al., 1993) Between 4 and 6 Hz, the tremor is often unilateral and prominent in the distal part of the limb. It often occurs in the lips, jaw, and legs rather than in the neck and head (Jankovic, 2008).
Rigidity		Resistance increases during passive movement of the limb (flexion, extension, or rotation of the joint such as neck, shoulders, hips, wrists, ankles) (Jankovic, 2008).
Bradykinesia		Difficulty in planning, initiating and executing sequential or simultaneous actions. Slow down and reduce amplitude when performing rapid, repetitive, alternating hand movements (finger tapping, forward-superior hand tilt) and heel tapping. Most correlated with the degree of dopamine deficiency (Vingerhoets et al., 1997).
Postural balance disorders		Caused by loss of postural reflexes, it is usually a manifestation of advanced Parkinson’s disease (Jankovic, 2008). Postural instability (and freezing of gait) is the most common cause of falls (Williams et al., 2006).
Non-motor symptoms		
Sensory symptoms	Somatosensory dysfunction and pain	30–85% patients Paresthesia and numbness Pain: Arthritic or neurogenic distribution
	Visual disturbances	22–78% patients Visual Hallucinations
Autonomic symptoms	Olfactory loss	85% patients (Goldman and Postuma, 2014) No response to Parkinson’s drugs now (Morley and Duda, 2011; Choi et al., 2020)
	Sleep dysfunction	30–50% patients Insomnia Sleeping accompanied by talking, yelling, swearing, scratching, hitting, kicking, jumping, and other dramatic, forceful, and potentially injurious movements (RBD) (Gjerstad et al., 2007). Excessive daytime sleepiness (EDS) (Leite Silva et al., 2023) Restless Legs Syndrome (RLS) (Leite Silva et al., 2023) Possibly related to a decrease in hypocretin (orexin) neurons (Fronczek et al., 2007) (Thannickal et al., 2007)
	Orthostatic hypotension	70% patients (Palmiter, 2007) In the upright position, patients may present with dizziness, visual disturbances and cognitive deficits, which may precede loss of consciousness (Leite Silva et al., 2023)
Neuropsychiatric symptoms	Anxiety	40% patients (Leite Silva et al., 2023) Generalized anxiety disorder (GAD) and social phobia (Felice et al., 2016) Dopaminergic damage (Borgonovo et al., 2017)
	Apathy	60% patients A hypomotivational state (Nassif and Pereira, 2018) Dopaminergic denervation process or serotonergic degeneration (Leite Silva et al., 2023)
	Depression	30% patients (Jellinger, 1999) Guilt, sadness, lack of self-esteem and remorse (Chaudhuri et al., 2006) Dopaminergic damage (Borgonovo et al., 2017)
Cognitive impairment		84% patients (Jankovic, 2008) Can occur throughout Parkinson’s disease, dementia occurs late in the course of the disease

The primary clinical manifestations of PD, motor symptoms, exhibit discernible variations. Patients with PD can be categorized into tremor-predominant, rigid motor and mixed types based on their motor symptoms. The most prominent clinical symptoms of PD are resting tremor, rigidity, bradykinesia, and postural balance disorders. It is commonly believed that these classical motor symptoms result from degeneration of the nigrostriatal pathway and depletion of dopamine in the striatum (Gelpi et al., 2014; Schapira et al., 2017). The onset of motor signs in PD becomes evident when approximately half of the cells in the caudal substantia nigra have been lost (Fearnley and Lees, 1991). Therefore, the pharmacological treatment for motor symptoms in PD primarily focuses on dopamine (Armstrong and Okun, 2020). However, it is important to recognize that complications, such as dyskinesias, may emerge following a period of dopaminergic treatment (Armstrong and Okun, 2020).

While traditionally framed as a motor-centric disorder, PD unveils a diverse array of non-motor symptoms, emphasizing the significance of a detailed understanding of the condition. This heterogeneity encompasses a spectrum of manifestations, including rapid eye movement sleep behavior disorder (RBD), depression, autonomic dysfunction, orthostatic hypotension, cognitive impairments, and discomfort, collectively forming an intricate mosaic within the PD clinical spectrum (Pont-Sunyer et al., 2015). Rooted in peripheral system or limbic system neurodegeneration, these diverse non-motor symptoms transcend the traditional boundaries of PD, adding layers to its complexity (Gelpi et al., 2014; Schapira et al., 2017). Compared to motor symptoms, non-motor symptoms exhibit a prevalence that surpasses expectations, having a greater impact on patients and caregivers. Hyposmia or anosmia, which occurs in about 90% of people with PD, is listed by the Movement Disorder Society (MDS) criteria for PD as one of the four criteria that support a diagnosis of PD, illustrating the diverse facets shanping the PD narrative (Doty, 2012; Postuma et al., 2018; Tolosa et al., 2021). Although RBD has only about a 30–50% chance of occurring in PD, research has shown that over 90% of individuals with RBD symptoms eventually develop synuclein-related neurodegenerative diseases, including PD, underlines the intricate and diverse trajectories within the PD journey (Howell and Schenck, 2015; Galbiati et al., 2019). This diversity extends to cognitive realms, with varied forms of impairment influencing the trajectory of the disease and impacting the likelihood of developing dementia (Hely et al., 2008; Williams-Gray et al., 2013; Armstrong and Okun, 2020). These multifaceted layers, ranging from impulsive behaviors to obsessive-compulsive tendencies, add unique hues to the overall PD canvas, enriching the narrative with the diversity inherent in the condition (Miyasaki et al., 2007; Palmiter, 2007). However, despite the profound impact of these non-motor features on hospitalization, nursing home admissions, and the broader socio-familial fabric (Safarpour et al., 2015; Barone et al., 2017), their subtlety often renders them unnoticed during clinical consultations (Chaudhuri et al., 2010). This oversight stems from both patient unawareness of their association with PD and the diverse, elusive forms they may assume during onset, highlighting the imperative of recognizing and embracing the diversity inherent in the PD experience.

## 3 Family and sporadic PD

Approximately 15% of patients living with PD have a family history of the disease (Deng et al., 2018). Familial PD, also known as Mendelian or monogenic PD, is typically investigated when there is a high risk of developing the condition due to rare parental variants (Luth et al., 2014). The relative risk (RR) of having a first-degree relative with PD, compared to not having a first-degree relative with PD, ranged between 1.6 and 10.4 (Wirdefeldt et al., 2011). There is a stronger familial aggregation of early-onset PD compared to late-onset PD (Payami et al., 2002; Marder et al., 2003; Korchounov et al., 2004). Familial PD, in addition, has been reported to exhibit less cognitive impairment and a slower progression of dementia compared to sporadic PD (Dujardin et al., 2001; Inzelberg et al., 2004).

The sporadic form accounts for the majority of PD cases, and age remains the greatest risk factor for its development (Malpartida et al., 2021). However, it is worth noting that almost all cases of PD are likely to have detectable genetic effects, with the frequency and magnitude of the effects of the specific genetic variants involved varying in individual cases (Luth et al., 2014). However, it is not clear whether these mechanistic and pathological associations of clinical heterogeneity in hereditary PD also apply to sporadic. Certain genetic variants, such as SNCA, LRRK2, and GBA, are considered rare genetic variants with large effect sizes or low-penetrance genetic variants. These variants are known to be risk factors for sporadic diseases (Deleidi and Gasser, 2013; Nalls et al., 2014).

Interestingly, various variants of the same gene may all be associated with PD. For example, autosomal dominant familial PD is often caused by missense SNCA variations (p.A53T, p.G51D, p.A30P, and p.E46K) (Wittke et al., 2021). Conversely, common variants like SNCA rs356168, found in 40% of people of European heritage, have an effect on disease risk that is only slightly elevated (odds ratio ∼1.3) and do not significantly increase family risk (Nalls et al., 2019). Many families with mutations in the SNCA gene share overlapping pathology and clinical features, as evidenced by prominent cortical Lewy body formation and early onset of non-motor symptoms such as autonomic dysfunction and dementia. The early onset, rapid progression, and poor prognosis of these patients suggest that SNCA-associated mechanisms may be drivers of disease severity (Morris et al., 2024). Patients with PRKN mutations and predominantly mitochondrial dysfunction show a restricted pattern of cell loss, largely confined to the substantia nigra striata system, without the extensive pathological and non-motor features found in typical sporadic PD (Morris et al., 2024). Mutations in 11 genes associated with PD (SNCA, PINK1, PRKN, DJ1, ATP13A2, PLA2G6, FBXO7, LRRK2, CHCHD2, VPS35, and VPS13C) influence mitochondrial energy generation, reactive oxygen species production, mitochondrial biogenesis, and quality control, according to genetic research (Li et al., 2021). The utilization of high and low-risk alleles to distinguish between familial and sporadic diseases may have significant implications for clinical diagnosis, prognosis, and the advancement of genetic research (Luth et al., 2014). The biological mechanisms and therapeutic strategies may be highly relevant in individuals with a common genetic background. However, since PD is impacted by a variety of intricate elements, including environment and age, familial and sporadic PD are also subtypes that are targets for precision medicine.

## 4 Pathophysiology

Targeted gene-based therapies rely on genetic diagnosis, and valid serological biomarkers. Once a specific gene is involved in the pathological process, it is essential to identify characteristic blood disease markers for personalized treatment. For example, a study identified potential blood markers of PD through an integrated analysis of gene expression and DNA methylation data. This study confirmed the importance of these markers for the early identification, diagnosis, and treatment of PD (Wang et al., 2019).

Disease phenotypes are diverse, and the exact etiology has yet to be known. These processes may involve endosomal-lysosomal malfunction, inflammatory signaling, intracellular trafficking, dysregulation of mitochondrial homeostasis, and compromised systems linked to cell death mechanisms (Bandres-Ciga et al., 2020). As cellular processing is dynamic, neurodegeneration occurs in response to prolonged injury or stress, and various compensatory mechanisms are at work. Therefore, it is not possible to identify these pathways as acting independently or as a single pathway of neuronal death (Jankovic and Tan, 2020). The more likely scenario is that various pathophysiological processes intersect with each other to create a cascade of irreversible cellular damage that ultimately leads to disease (Jankovic and Tan, 2020). Each known pathological process is gradually being targeted for the treatment of diseases.

### 4.1 α-synuclein

The syndrome manifests itself as a result of progressive neuronal degeneration and increased abnormal α-synuclein protein (O'Keeffe and Sullivan, 2018). A-synuclein is a small 140-amino acid protein divided into three distinct regions: the N-terminal amphipathic region, the central hydrophobic region, and the C-terminal domain (Bayer et al., 1999; Giasson et al., 2001; Luth et al., 2014). The role of α-synuclein in the pathogenesis of PD has been controversial (Kalaitzakis et al., 2008). One hypothesis suggests that α-synuclein exists as a disordered protein or an unstructured monomer (Beyer, 2007; Fauvet et al., 2012; Bender et al., 2013). Others suggest that α-synuclein may exist as a tetramer and that it may destabilize the tetramer, resulting in a monomer (Jensen et al., 2011; Dettmer et al., 2013). Its physiological role is believed to be important for the aggregation of synaptic vesicles, efflux, and recycling through lectin-mediated endocytosis (Bayir et al., 2009; Dettmer et al., 2013; Burré et al., 2018). A-synuclein is implicated in several processes, including neurotransmission, lysosomal dysfunction, mitochondrial dysfunction, and activation of the neuroimmune response (Burré et al., 2018; Ye et al., 2023). It aggregates to create protein inclusions inside the Lewy bodies and Lewy neurites. Lewy’s lesions are assumed to progress in the following manner: they are believed to start in the caudal brainstem or the olfactory bulb, and move via the limbic areas, upper brainstem, and neocortex (Braak et al., 2002). The development of PD’s clinical phenotype is tightly linked to this pattern.

A-synuclein protein is widely regarded as an essential component in the pathogenesis of PD, and several pathogenic genes can influence the aggregation of α-synuclein protein, hence contributing to the development of PD (Ye et al., 2023). Apart from SNCA, PD is also caused by other genes including GBA, LRRK2, and MAPT, which interfere with the regular functioning of α-synuclein proteins. Mutations in LRRK2 might potentially intensify the harmful consequences of α-synuclein proteins by impacting the autophagy-lysosome system, mitochondrial operations, phosphorylation of RAB protein, or interactions between 14 and 3-3 proteins (Cresto et al., 2019). GBA genes regulate the activity of β-glucocerebrosidase, which modifies glycosphingolipid balance and causes pathological alterations including aberrant α-synuclein aggregation (Vijiaratnam et al., 2021).

*In vitro* testing of proteins that act as biomarkers for PD improves the accuracy of early diagnosis of the disease, clarifies subtypes and accelerates clinical trials. Recent studies have shown that seeded amplification assays (SAAs) are capable of detecting αSyn-related aggregates in brain homogenates (BHs) and cerebrospinal fluid sample (Majbour et al., 2022). Real-time shock-induced conversion (RT-QuIC) and protein misfolding cyclic amplification are both SAA (Shahnawaz et al., 2020; Orrù et al., 2021; Mammana et al., 2024). A meta-analysis demonstrated its ability to accurately and reliably diagnose Lewy body diseases such as PD (Wang et al., 2022). Studies have shown that α-synuclein-specific analyses performed in cerebrospinal fluid (CSF) can differentiate patients with PD from healthy controls with a high degree of sensitivity and specificity (Siderowf et al., 2023).

It has long been known that α-synuclein plays a key role in PD therapy options. Targets may be divided into three categories: directly targeting α-synuclein itself; upstream variables that may cause pathological α-synuclein alterations; or downstream pathways linked to the spread of pathogenic α-synuclein changes or potentially stimulating neural compensatory responses (Vijiaratnam et al., 2021). These findings laid the groundwork for comprehending the disease’s pathophysiology and its treatment goals, which are mainly enhancing clearance and preventing aggregation. Clinical trials are presently being conducted on a number of these tactics (Jankovic and Tan, 2020; Vijiaratnam et al., 2021). Many of the relevant therapeutic approaches will be detailed below in the presentation of the SNCA gene.

### 4.2 Lysosome

The lysosome can act as a regulatory hub for homeostasis through endocytosis, phagocytosis, or autophagy (Ballabio and Bonifacino, 2020; Ye et al., 2023). It can also exchange content and information and establish membrane contact sites to communicate with other cellular structures (Ballabio and Bonifacino, 2020). Remarkably, lysosomes are closely related to α-synuclein in the pathogenic process of PD (Nguyen et al., 2019; Ye et al., 2023). In the abnormal state, autophagy-lysosomes hinder the clearance of αSyn and facilitate its aggregation, pathological spread, and cytotoxicity. Conversely, toxic αSyn species disrupt the biogenesis and function of lysosomes. This is a positive feedback loop that eventually leads to the abnormal death of dopaminergic neurons and the onset of PD (Horowitz et al., 2022). Many genes related to PD play a role in encoding proteins that are associated with lysosomes. These proteins include lysosomal membrane proteins (e.g., TMEM175), lysosomal enzymes (e.g., GBA), and regulators of endosomal-lysosomal trafficking (e.g., LRRK2, VPS35).

### 4.3 Mitochondria

Under normal physiological conditions, mitochondria are the most important organelles that provide energy to neurons. Mitochondrial dysfunction is strongly associated with both sporadic and familial PD (Rocha et al., 2018). Abnormal mitochondrial dynamics, biogenetic damage, complex I inhibition of the electron transport chain (ETC), and increased reactive oxygen species (ROS) are particularly noteworthy (Winklhofer and Haass, 2010; Ryan et al., 2015). Mitochondria produce ATP through oxidative phosphorylation (OXPHOS), which consists of an electron transport chain (ETC) and ATP synthase. Supplying carbon fuels to the tricarboxylic acid cycle (TCA) produces electron donors NADH and FADH2, which provide electrons for mitochondrial complexes I-V (MCI-MCV). These complexes are transmembrane proteins located in the inner mitochondrial membrane (Subramaniam and Chesselet, 2013; Greene et al., 2022). Intracellular reactive oxygen species mainly originate from mitochondrial complex I (MCI) and mitochondrial complex III (MCIII) of the electron transport chain (ETC). Abnormalities in the ETC not only result in the loss of mitochondrial biological functions but also cause oxidative stress and increase the susceptibility of neurons to excitotoxic damage, ultimately leading to PD (Zuo and Motherwell, 2013).

Mitochondrial homeostasis is connected to the majority of genes linked to PD, including SNCA (PARK1/4), PRKN (PARK2), PINK1 (PARK6), DJ-1 (PARK7), LRRK2 (PARK8), ATP13A2 (PARK9), PLA2G6 (PARK14), FBXO7, VPS35, CHCHD2, and VPS13C (Monzio Compagnoni et al., 2020; Toffoli et al., 2020). Through a variety of processes, including mitochondrial morphology, quality control, biogenesis (fission/fragmentation), and processes like the electron transport chain (ETC) and reactive oxygen species (ROS) release, these genes contribute to the maintenance of mitochondrial homeostasis (Li et al., 2021). Due to the unique structure of α-synuclein, it has an affinity for the mitochondrial membrane and tends to accumulate there (Subramaniam and Chesselet, 2013). Once this occurs, it will also contribute to abnormal mitochondrial function. The fundamental process of the reciprocal association between α-synuclein and mitochondrial dysfunction could offer fresh perspectives on the etiology of PD and potential avenues for treatment (Rocha et al., 2018). The proteins encoded by PRKN, PINK1, DJ-1, LRRK2, and FBXO7 are closely related to α-synuclein (Li et al., 2021). Excessive α-synuclein accumulation is caused by mutations in LRRK2, ATP13A2, PLA2G6, VPS35, CHCHD2, and VPS13C. α-synuclein is closely related to proteins encoded by PRKN, PINK1, DJ-1, LRRK2, and FBXO7 (Li et al., 2021). Mutations in ATP13A2, LRRK2, VPS13C, VPS35, CHCHD2, and PLA2G6 cause an increase in the buildup of α-synuclein (Li et al., 2021).

With the growing understanding of mitochondrial homeostasis and the role of mitochondrial damage in PD, several potential therapeutic approaches have been increasingly validated. Small molecule activators of parkin and PINK1 may be one of these therapeutic targets (Malpartida et al., 2021). Currently, kinetin triphosphate (KTP) and other small molecules act as activators of PINK1 by directly expressing highly soluble and cell-permeable recombinant Parkinson’s proteins. This approach aims to protect neurons from toxins and α-synuclein damage, while also exploring the potential of bioavailable KTP precursors (Hertz et al., 2013; Lambourne and Mehellou, 2018; Chung et al., 2020). Moreover, several deubiquitinases (DUBs), including USP30, USP8, USP14, USP15, and USP35, regulate mitosis by antagonizing parkin activity. Therefore, inhibitors targeting these DUBs may be a promising area of research (Durcan et al., 2014; Chakraborty et al., 2018; Harper et al., 2018; Teyra et al., 2019). In addition, several studies have shown that LRRK2 kinase inhibitors, nicotinamide riboside, and the repositionable drug ursodeoxycholic acid can correct or enhance mitochondrial function (Schöndorf et al., 2018; Bonello et al., 2019; Carling et al., 2020; Wauters et al., 2020).

### 4.4 Neuroinflammation

Although it is uncertain whether neuroinflammation promotes or prevents neurodegeneration, there is sufficient evidence to prove that immune factors play an important role in the pathogenesis of PD. Cellular and humoral immunity can mediate the immune response, and significantly elevated levels of complement, cytokines (such as IL-1, IL-2, IL-6, and TNF), NO, and reactive oxygen species (ROS) have been observed in the substantia nigra and cerebrospinal fluid (CSF) of patients with PD (Liu et al., 2003). Among them, brain immune cells, especially microglia, play a crucial role in driving the disease process. Microglia activation is often discussed as a double-edged sword for tissue homeostasis. On one hand, microglia activation is required to remove apoptotic debris from dopamine neurons. However, on the other hand, it results in the excessive production of ROS, cytokines, and chemokines due to the direct stimulation of α-synuclein and indirect inflammatory signals. A-synuclein-induced microglia activation generates a burden of reactive oxygen species (ROS) that is particularly harmful to dopamine neurons. This effect is exacerbated in neurons that already have mitochondrial dysfunction, and it may contribute to either dopamine neuron dysfunction or cell death. Autoantibodies against antigens associated with the pathogenesis of PD have been identified in several studies. This confirms that immune factors can be the cause of PD development, not just the process. Triggers of the neuroinflammatory response, as part of the pathological process of a disease, may include protein aggregates (such as α-syn and amyloid β), dysregulation of inflammatory pathways (associated with aging or genetic susceptibility), and pathogens (bacterial or viral infections) (Deleidi and Gasser, 2013).

The expression of numerous genes implicated in PD is not exclusive to neurons but is also highly expressed in the immune system (Allen et al., 1997; Hakimi et al., 2011; Deleidi and Gasser, 2013; Guilhem de Lataillade et al., 2023). Some of the genes that regulate immune function by encoding proteins include LRRK2, SNCA, DJ1, GBA, PRKN, and PINK1 (Magistrelli et al., 2022; Tansey et al., 2022). Not only can LRRK2 and GBA directly influence the inflammatory process by being highly expressed in immune cells, but they can also trigger an inflammatory response due to their roles in autophagy and lysosomal function (Orenstein et al., 2013). Anti-inflammatory medications do not offer neuroprotection in the latter stages of PD, even though inflammation plays a major role in the onset and progression of the disease (Aisen et al., 2003; Cudkowicz et al., 2006). In addition, the lack of disease biomarkers has impeded research on anti-inflammatory medications at the prodromal stage of the illness (Deleidi and Gasser, 2013). Although immunomodulatory drugs have not been rigorously demonstrated in clinical pilot studies, immunomodulatory interventions have shown some superiority when used in combination with other neuroprotective agents. For instance, minocycline has been evaluated in PD patients and experimental models, exhibiting its anti-inflammatory and neuroprotective qualities (Plane et al., 2010). Since genes related to PD play a significant role in the inflammatory response process, there is also great potential for studying precision therapeutic genes involved in inflammatory pathways for the treatment of PD (Deleidi and Gasser, 2013).

## 5 Genetics

Our understanding of the genetic origins and risk variations of PD is rapidly advancing due to recent advancements in high-throughput genomic analysis and bioinformatics. We need to answer not only how genes affect disease mechanisms, but also how disease-associated genetic variants affect genes (Ye et al., 2023). Ninety independent variations in 78 genomic areas associated with PD have been identified via a meta-analysis of genome-wide association studies (GWAS). However, little is known about the processes by which these variants affect the development of PD (Farrow et al., 2022). For PD, autosomal dominant, recessive, and non-Mendelian types are thought to represent the main inheritance patterns. Thus far, autosomal dominant genes for PD have been discovered as SNCA, Leucine-Rich Repeat Kinase 2 (LRRK2), Vacuolar protein sorting-35 (VPS35), and eukaryotic translation initiation factor 4γ (EIF4G1). The genes linked to autosomal recessive PD include Parkin (PARK2), PTEN-induced kinase (PINK1), Daisuke-Junko-1 (PARK7), phospholipase A2, group VI (PLA2G6), F-box only protein 7 (FBXO7), and spastic paraplegia 11 (SPG11). Moreover, the inclusion of some non-Mendelian loci and disorders that do not follow the classic pattern of PD remains somewhat unclear. PD with a Mendelian, monogenic variant affects about 5–10% of PD patients (Deng et al., 2018; Chan, 2022). Mutations in genes such as SNCA, PRKN, PINK1, DJ-1, LRRK2, and VPS35 have been found to cause the deletion of dopamine (DA) neurons (Li et al., 2021). A genome-wide association study (GWAS) confirmed several known pathogenic genes related to PD, including SNCA, GBA1, LRRK2, and MAPT (Chang et al., 2017). Table 2 lists the mutations linked to monogenic forms of PD, but excludes loci without known causal genes. Much progress has been made in understanding the relationship between genetic variables and diseases, as well as in linking genes and pathways in Mendelian and non-Mendelian disorders (Lubbe and Morris, 2014). Directly targeting proteins affected by single-gene mutations in PD provides a strategy for expanding treatment to patients with genetic connections.

**Table 2:** Parkin Genes of PD.

Mutations	Genes	Chromosomal position	Inheritance pattern	Pathology	Major manifestations	References
PARK1(MIM 168601)	SNCA	4q21–22	AD	Lewy bodies	Early onset, rigidity, cognitive impairment	Polymeropoulos et al., (1997), Mizuno et al. (2008), Lesage and Brice (2009), and Caccappolo et al. (2011)
PARK2(MIM 602544)	PRKN	6q25.2–27	AR	Tau pathology	Early onset, no neuropsychological impairments,classic motor symptoms	Inzelberg and Polyniki (2010) and Caccappolo et al. (2011)
PARK4	SNCA	4q21–22	AD	Lewy bodies	Marked dementia and frequent dysautonomia	Lesage and Brice (2009)
PARK6	PINK1	1p35-36	AR	Lewy bodies	Early onset, psychiatric features	Valente et al. (2001)
PARK7	DJ-1	1p36	AR	Unknown	Early onset, psychiatric symptoms	Dekker et al. (2003)
PARK8(MIM 607060)	LRRK2	12q12	AD	Lewy bodies	Early onset, classic PD, dementia	Funayama et al. (2002), Lesage and Brice (2009), and Inzelberg and Polyniki (2010)
PARK9	ATP13A2	1p36	AR	Lysosomes	Early onset, cognitive impairment	Di Fonzo et al. (2007) and Mizuno et al. (2008)
PARK14	PLA2G6	22q13.1	AR	Iron accumulation on MRI	Early onset, cognitive impairment, dystonia	Paisan-Ruiz et al. (2009)
PARK15	FBXO7	22q12–13	AR	Modulate proteasome functions	Early onset, classic PD	Zhou et al. (2018)
PARK17	VPS35	16q11.2	Unknown	Lysosomal	Late onset, classic PD	Zimprich et al. (2011)
PARK19(MIM 615528)	DNAJC6	1p31.3	AR	Synaptic vesicles endocytosis and trafficking	Early onset, slow disease progression, dystonia	Olgiati et al. (2016) andNg et al. (2020)
PARK20	SYNJ1	21q22.11	AR	Tau pathology	Early onset, seizures	Hardies et al. (2016)
PARK21	DNAJC13	3q22.1	AD	Lewy bodies	Classic PD	Vilariño-Güell et al., (2014) and Gagliardi et al. (2018)
PARK23	VPS13C	15q22.2	AR	Mitochondrial Dysfunction, Lewy bodies	Early onset, rapid progression, classic PD, early cognitive decline	Lesage et al. (2016) and Schreglmann and Houlden (2016)

Classic PD refers to symptoms that resemble sporadic PD. PD, Parkinson’s disease; AD, autosomal dominant; AR, autosomal recessive.

### 5.1 SNCA (PARK1)

SNCA (NG_011851), the α-synuclein gene, its mutations, locus multiplication, promoter polymorphisms, and rare missense mutations are closely related to syndromes, particularly motor symptoms and cognitive decline (Shulman et al., 2011). SNCA genomic triplication has been reported to cause autosomal-dominant early-onset PD with dementia (Chartier-Harlin et al., 2004; Farrer et al., 2004). The missense mutations A30P, E46K, and A53T in the N-terminal region of the α-synuclein protein are associated with familial PD (Table 3; Chartier-Harlin et al., 2004). Significant loss of hippocampal CA2/3 neurons was found in brains with SNCA missense mutations (Waters and Miller, 1994; Muenter et al., 1998; Gwinn-Hardy et al., 2000; Spira et al., 2001). Dementia may result from a single gene over-replication of SNCA, which may be linked to the start, course, and severity of PD (Chartier-Harlin et al., 2004). Genetic research has demonstrated that idiopathic PD is associated with genetic diversity within the α-synuclein promoter (Farrer et al., 2001). Therefore, reducing the production of α-synuclein, inhibiting its aggregation, and increasing its clearance may be a very promising therapeutic approach.

**Table 3:** Variants of SNCA.

Variant and amino acid sequence	Inheritance pattern	Age of onset	Pathology	Major manifestations	References
c.G188A p.E46K	AD/family	Range: 50–65 years	Lewy bodies	Parkinsonian motor symptoms responsive to levodopa with early onset, early cognitive impairment, sleep disorders and autonomic dysfunction and dystonia.	Zarranz et al. (2004) and Somme et al. (2011)
c.G209A p.A53T	AD/family	Average: 40.8 years	Mutant alpha-synuclein results in impaired vesicular dopamine storage, triggering free radical overproduction and mitochondrial dysfunction, leading to cell death.	Classic PD with good response to levodopa, rapidly progressive course, and cognitive impairment.	Polymeropoulos et al. (1997), Lotharius et al. (2002), and Xiong et al. (2016)
c.G88C p.A30P	AD/family	Average: 60 years	Lewy bodies	Bradykinesia and cognitive impairment, responsive to levodopa.	Krüger et al. (1998) and Seidel et al. (2010)
c.C152A p.G51D	AD/family	Range: 19–71 years	A-synuclein inclusions and small numbers of GCI-like inclusions.	Variable levodopa response, dementia, persistent visual hallucinations and autonomic dysfunction.	Kiely et al. (2015) and Lau et al. (2023)

For each variant, the nucleotide and amino acid change is provided along with Inheritance patterns, known kindreds, clinical and pathological features, and relevant references.GCI, glial cytoplasmic inclusion; AD, autosomal dominant; PD, Parkinson’s disease; Classic PD refers to symptoms that resemble sporadic PD.

There have been many attempts at therapies targeting SNCA. First of all, RNA interference (RNAi) technologies are intended for the targeted suppression of α-synuclein synthesis before its polymerization (Toffoli et al., 2020). When short hairpins and small interfering RNA (siRNA) were injected into the striatum and hippocampus of mouse and primate models, the production of α-synuclein was reduced even after three weeks. This demonstrates the potential therapeutic impact of RNA-based therapy on α-synuclein-associated diseases (Sapru et al., 2006; Lewis et al., 2008; McCormack et al., 2010). Alternatively, the use of β2-adrenergic receptor (β2AR) agonists, which regulate gene transcription through histone 3 lysine 27 acetylation, reduces the transcription of the α-synuclein gene (Mittal et al., 2017; Gronich et al., 2018).

Neurotoxicity occurs due to misfolding or aggregation of α-synuclein proteins and the formation of Lewy bodies (Toffoli et al., 2020). A small antibody fragment, known as an antibody endosome, binds to intracellular A-SYN, preventing its oligomerization. This fragment can be delivered either as a protein or a gene (Chatterjee et al., 2018). Despite this, several other therapies, such as the small molecule NPT200-11 and the biological compound NPT088, are currently in early clinical trials to inhibit α-synuclein aggregation (Levenson et al., 2016; Krishnan et al., 2017).

Thirdly, increased α-synuclein clearance can be achieved through immunotherapy and activation of autophagic pathways (Toffoli et al., 2020). The two forms of anti-α-synuclein immunotherapy used in clinical programs include passive immunization, which involves the use of specific antibodies against α-synuclein, and active immunization, which involves the injection of modified α-synuclein to stimulate the production of endogenous antibodies (Toffoli et al., 2020). In particular, there is a growing number of active and passive immunization methods being developed. Humanized IgG1 monoclonal antibodies such as ABBV-0805, RO7046015/PRX002, and BIIB-054 are a few examples (McFarthing and Simuni, 2019; Zella et al., 2019). Several obstacles exist when using immunotherapy to increase A-SYN degradation, including the possibility of off-target reactions, the requirement for frequent administration, the absence of an immune response to active treatments, and the lack of certainty about whether the restricted antibody penetration into the central nervous system (CNS) is sufficient for meaningful A-SYN elimination (Lindström et al., 2014). Autophagy has been recognized as one of the major pathways for degrading intracellular A-SYN aggregates (Xilouri et al., 2016). The neuroprotective effects of the autophagy enhancers rapamycin and lithium are currently being investigated for their ability to reduce A-SYN aggregates (Webb et al., 2003; Crews et al., 2010; Decressac et al., 2013; Forlenza et al., 2014). However, neither lithium nor rapamycin are appropriate for long-term, high-dose usage since they both have adverse effects, lack specificity, and interact with several cellular processes (Toffoli et al., 2020). Interestingly, the inhibitor of the mitochondrial pyruvate carrier (MPC), MSDC-0160, and an anti-cancer drug called Nilotinib, act as therapeutic approaches to reduce protein aggregation (Ghosh et al., 2016). Finally, it is worth noting that the mechanisms of SNCA pathogenesis are still under debate. Therefore, large-sample, high-quality clinical and preclinical trials will continue to be the main focus of future research.

### 5.2 LRRK2 (PARK8)

Unlike SNCA, measures of LRRK2 expression do not affect the disease phenotype (Lesage and Brice, 2009). LRRK2 is one of the genes associated with autosomal dominant PD and belongs to the ROCO family of proteins. It consists of five major functional domains: the Roc structural domain (Ras in complex proteins), the leucine-rich repeat sequence (LRR), the COR structural domain (located at the C-terminus of the Roc), the WD40 structural domain, and the TyrKc structural domain (catalyzed by tyrosine kinases) (Bosgraaf and Van Haastert, 2003; Zimprich et al., 2004). Some early studies have shown that PD caused by LRRK2 mutations is difficult to distinguish from sporadic and idiopathic PD (Adams et al., 2005; Ross et al., 2006). When compared to patients with idiopathic PD, the most prevalent cause of autosomal-dominant PD (Gly2019Ser in LRRK2) was linked to a decreased likelihood of cognitive impairment and olfactory hyposmia (Healy et al., 2008). There are additional LRRK2 variants that significantly alter the risk of PD (Table **4**). The motor phenotype of LRRK2 PD is thought to progress slowly (Healy et al., 2008). However, a previous series of studies revealed differences in the non-motor symptoms between individuals with PD who carry the LRRK2 mutation and those who do not. Some studies suggest that Parkinson’s disease (PD) related to LRRK2 may exhibit milder cognitive symptoms (Aasly et al., 2005; Lesage et al., 2005; Healy et al., 2008; Ben Sassi et al., 2012; Srivatsal et al., 2015). According to other research, there is no difference in Minimum Mental State Examination (MMSE) scores between those with and without Parkinson’s disease (PD) who carry the LRRK2 mutation (Belarbi et al., 2010; Alcalay et al., 2010a; Shanker et al., 2011; Ben Sassi et al., 2012; Trinh et al., 2014). Regarding the milder cognitive symptoms found in LRRK2 mutations, a study suggests that it may be related to the prevalence of Lewy body-negative cases in LRRK2 cohorts (Srivatsal et al., 2015).

**Table 4:** Variants of LRRK2.

Variant and amino acid sequence	Inheritance pattern	Age of onset	Pathology	Major manifestations	References
c.6055G > A p.G2019S	AD/both familial and sporadic	Both early and late onset	Increase kinase activity	Earlier age at onset motor symptoms, depression and hallucinations	Aasly et al. (2005), Nichols et al. (2005), Goldwurm et al. (2006), Healy et al. (2008), Lesage and Brice (2009), Belarbi et al. (2010), Alcalay et al. (2010b), Shanker et al. (2011), Thaler et al. (2012), and Cookson (2015)
c.7153G > A p. G2385R	Sporadic	NA	Increases kinase activity	Motor scores worsened more rapidly	Rudenko et al. (2012) and Marras et al. (2016)
c.4883G > C p.R1628P	AD/both familial and sporadic	NA	COR, increases kinase activity	Motor scores worsened more rapidly	Marras et al. (2016) and Zhang et al. (2017)
c.4939 T > A p.S1647T	Familial	NA	COR	Motor scores worsened more rapidly	Marras et al. (2016)
c.4321C > T/G/A p.R1441C/G/H/S	AD	NA	Decrease GTPase activity	NA	Paisán-Ruíz et al. (2004)
c.6059 T > C p.I2020T	AD	NA	Increase kinase activity	NA	Ray et al. (2014)
c.4309A > C p.N1437H	AD/both familial and sporadic	Early onset	ROC, decrease GTPase activity	Early development of marked motor fluctuations and dyskinesias.	Puschmann et al. (2012) and Cookson (2015)
c.5096A > G p.Y1699C	AD	NA	Strengthens the intra-molecular ROC: COR interaction, decrease GTPase activity	NA	Daniëls et al. (2011) and Cookson (2015)
c.1464A > T p.A419V(rs34594498)	Increased sporadic PD risk	Early onset	NA	Cognitive impairment	Li et al. (2015)
c.4937 T > C p.M1646T	NA	NA	Increase kinase activity	NA	Sosero et al. (2021)

For each variant, the nucleotide and amino acid change is provided along with Inheritance patterns, known kindreds, clinical and pathological features, and relevant references.AD, autosomal dominant; COR, C-terminal of Ras; ROC, Ras of complex proteins; NA, not applicable.

The pathogenicity of LRRK2 may be related to the GTPase and kinase activities of the gene’s protein (Toffoli et al., 2020). Abnormally elevated intracellular and extracellular LRRK2 protein kinase activities are strongly associated with PD pathogenesis (West et al., 2005; Sheng et al., 2012). Efforts to reverse this pathological process have primarily focused on reducing kinase activity through the use of kinase inhibitors. Studies have ranged from non-selective kinase inhibitors that are unable to cross the blood–brain barrier to a new generation of selective LRRK2 inhibitors that can cross the blood–brain barrier, such as HG10-102-01 (Choi et al., 2012), JH-II-127 (Hatcher et al., 2015), and TAE684 (Zhang et al., 2012). Subsequently, MLi-2 (Fell et al., 2015; Scott et al., 2017) and PFE360 (Andersen et al., 2018) were developed, which demonstrated excellent performance in terms of inhibiting LRRK2 kinase activity, selectivity, pharmacokinetics, and safety. Several preclinical and clinical studies have been conducted progressively on this basis (Choi et al., 2012; Zhang et al., 2012; Fell et al., 2015; Hatcher et al., 2015; Scott et al., 2017; Andersen et al., 2018). Denali Therapeutics demonstrated that the inhibitor may act on the lysosomal pathway of PD by testing DNL201 and BIIB122 in both healthy volunteers and patients with PD (Jennings et al., 2022, 2023). Although the LRRK2 gene pathways appear promising for the precise treatment of PD, several challenges still exist, such as toxicity and the absence of specific biomarkers. If people with sporadic PD share the same processes or whether these prospective therapies may be more broadly applicable are still unclear issues (Di Maio et al., 2018).

### 5.3 MAPT

It is well known that protein aggregation and inclusion formation are facilitated by the microtubule-associated protein tau (MAPT). PD autosomal dominant variants have been associated with mutations in MAPT (Simón-Sánchez et al., 2009). Numerous investigations have revealed a strong correlation between cognitive impairment in PD patients and the advancement of dementia and mutations in the MAPT gene (Goris et al., 2007; Williams-Gray et al., 2009). In contrast, the strong correlation between MAPT and cognitive decline was found to be highly dependent on age (Goris et al., 2007; Williams-Gray et al., 2009). Notably, MAPT and SNCA have synergistic effects in the pathogenesis of PD (Goris et al., 2007; Clarimón et al., 2009; Williams-Gray et al., 2009; Setó-Salvia et al., 2011; Morley et al., 2012; Nombela et al., 2014; Winder-Rhodes et al., 2015; Paul et al., 2016). However, due to the polymorphism of MAPT loci and its genetic imbalance, the mechanism by which MAPT causes PD is still unclear.

Notably, Roberto et al. demonstrated that reduced levels of MAPT-AS1 and the presence of the MAPT H1 haplotype may combine to cause high tau-IRES activity and increase the risk of PD by disrupting tau protein homeostasis (Simone et al., 2021). Therefore, reversing this pathological change may be the key to targeting the MAPK pathway for future therapeutic purposes.

### 5.4 GBA

Mutations in the glucocerebrosidase (GBA) gene have been identified as risk factors for PD, with Lewy bodies being implicated in the pathogenic processes of these mutations (Clark et al., 2009; Neumann et al., 2009; Sidransky et al., 2009; Alcalay et al., 2012; Smith and Schapira, 2022). It is now well-established that GBA mutations occur in both familial and sporadic PD (Winder-Rhodes et al., 2013). The most prevalent single mutation linked to sporadic PD is GBA heterozygous mutations, which are five times higher in PD patients than in the general population (Sidransky et al., 2009). The risk of PD is modified by several frequent GBA mutations (Table 5). Non-motor symptoms, a more severe clinical history, and an early onset are typical features of GBA mutation-associated PD. It primarily leads to cognitive impairment or dementia (Clark et al., 2007; Gan-Or et al., 2008; Mitsui et al., 2009; Alcalay et al., 2010b, 2012; Chahine et al., 2013; Oeda et al., 2015; Mata et al., 2016). In the meanwhile, a research revealed that compared to controls, PD patients with GBA mutations experienced a faster progression of motor symptoms (Brockmann et al., 2015). Individuals with PD who have GBA mutations respond well to levodopa and do not develop progressively severe motor impairments during the course of the disease (Setó-Salvia et al., 2012). One study, which explores the various non-motor characteristics in patients with GBA-PD, suggests that the severity of the GBA variant may be responsible for different phenotypic features. Therefore, it may be essential to stratify patients with PD based on the severity of the GBA variant to select appropriate treatments (Ren et al., 2022).

**Table 5:** Variants of GBA.

Variant and amino acid sequence	Inheritance pattern	Age of onset	Pathology	Major manifestations	References
c.1448 T > C p.L444P	Sporadic	Average: 47 years	Lewy bodies	Dementia	Clark et al. (2007)
c.1226A > G p.N370S	Sporadic	Early onset	Lewy bodies	Dementia	Clark et al. (2007)
c.1223C > T p.T369M	Sporadic	NA	Lewy bodies	Dementia	Clark et al. (2007)
c.1604G > A p.R496H	Sporadic	NA	Lewy bodies	Dementia	Clark et al. (2007)

For each variant, the nucleotide and amino acid change is provided along with Inheritance pattern, known kindreds, clinical and pathological features, and relevant references.NA, not applicable.

Mechanisms of GBA gene mutations associated with PD lesions may include α-synuclein protein deposition, mitochondrial dysfunction, and inhibition of autophagy. Genetic variation of the GBA gene, which encodes the lysosomal enzyme β-glucosylceramide (GCase), has been associated with PD (Sidransky et al., 2009). β-glucocerebrosidase is synthesized, folded, and delivered to the lysosomes under normal conditions. Nevertheless, the endoplasmic reticulum retains the mutant β-glucocerebrosidase, which causes abnormal vesicular trafficking, a reduction in lysosomal concentration, and α-synuclein aggregation (Do et al., 2019; Vijiaratnam et al., 2021). Based on the expression of the GBA gene product, several therapeutic approaches have been attempted, including Enzyme Replacement Therapy (ERT), which involves regular infusions of GCase (Stojkovska et al., 2018). Though not very successful, other approaches have been investigated, including chaperoning GCase to the lysosome, substrate reduction treatment, and using viral vectors to insert wild-type GBA1 alleles into the genomes of GBA1 mutation carriers (Morabito et al., 2017). Apart from these endeavors, there exists an additional conjecture. When a misfolded GCase protein is unable to be refolded by ER chaperones, it experiences ER-associated degradation (ERAD). The unfolded GCase proteins are then redirected to the proteasome for degradation, which promotes ER stress and activates the unfolded protein response (UPR) (Gegg et al., 2022). Misfolded GCase proteins will block CMA, and the activated Unfolded Protein Response (UPR) will disturb intracellular calcium homeostasis. All of these factors will speed up the progression of PD (Schöndorf et al., 2014; Kilpatrick et al., 2016; Kuo et al., 2022). Therefore, therapeutic strategies aimed at stabilizing and refolding misfolded GCase proteins, thereby relieving ER stress, maybe another attractive option for the treatment of PD (Menozzi et al., 2023). Nevertheless, the pathological mechanisms of GBA-mutant PD are still unclear, and as a result, no effective treatment has been found to date.

## 6 Age

Normal aging occurs due to an array of elements that include genomic instability, telomere attrition, loss of proteostasis, epigenetic modifications, mitochondrial dysfunction, cellular senescence, stem cell fatigue, unregulated nutrition sensing, and altered intercellular communication. Precision therapy must therefore take age-related PD into account (Hou et al., 2019). With a median age at onset of 60 years old, age is the most important risk factor for PD (Ascherio and Schwarzschild, 2016; Simon et al., 2020). Age has a substantial effect on PD symptoms, especially when it comes to motor fluctuations and cognitive function. In general, younger patients are more likely to experience significant motor benefits from levodopa therapy (Berg et al., 2021). In contrast, dementia occurs almost exclusively in older patients, and cognitive impairment is more closely related to age than to the duration of the disease (Anang et al., 2014). However, the age-related pathology of PD has not yet been clarified. Some studies suggest age-related dysfunctions in proteostatic mechanisms may lead to α-synuclein folding errors, leading to a diffuse rather than a ‘mitochondrial’ pattern of selective substantia nigra degeneration. New potential targets for intervention in PD may emerge from the significance of age-related DNA damage and repair (Li et al., 2022).

## 7 Lifestyle and environment

Underlying genetic factors, lifestyle choices, and environmental influences, along with their interactions, play crucial roles as both causative and therapeutic factors in PD at different stages. Exercise, smoking, alcohol consumption, and the consumption of dairy products and coffee are the main lifestyle factors that influence disease (Miyake et al., 2010; Ascherio and Schwarzschild, 2016; Collaborators, 2019). Interestingly, there is considerable evidence that smoking, caffeine consumption, and moderate alcohol consumption reduce the risk of PD by about 50% (Shaltouki et al., 2018; Jankovic and Tan, 2020). A study by Yoshihiro Miyake and his colleagues showed a significant additive interaction between the LRRK2 Gly2385Arg SNP and smoking in relation to the risk of sporadic PD (Miyake et al., 2010). Higher serum urate, history of melanoma, type 2 diabetes mellitus, and head trauma are other reported associations with ibuprofen use (Ascherio and Schwarzschild, 2016). Finally, patients with higher levels of education exhibited superior baseline motor and cognitive functioning in comparison to those with lower levels of education (Lee et al., 2019).

The neuroprotective benefits from different lifestyle decisions in PD are uncertain. Nicotine may have a protective effect on dopaminergic neurons because it stimulates the release of dopamine (Jankovic and Tan, 2020). Caffeine may exert neuroprotective effects by blocking adenosine A2a receptors (Jankovic and Tan, 2020). Uric acid may have neuroprotective properties due to its ability to act as a free radical scavenger. Although research on this topic is still ongoing, various lifestyles interact with each other, and the research methodology makes it difficult to draw definitive conclusions.

Environmental factors can interact with genetic factors, contributing to the diversity observed in Parkinson’s disease. Scientists are finding out that certain genes can make someone more likely to get PD, but the things they are exposed to in their environment can also affect this. Things like pesticides, head injuries, and certain toxins from industries might play a role. Pesticides can specifically affect the substantia nigra and associated brainstem nuclei. This can result in a purely motor phenotype, with dementia appearing later in the disease (Elbaz et al., 2004; Berg et al., 2021). Besides, exposure to environmental pesticides enhances the immune response in individuals carrying the HLA-DR variant and increases the risk of developing the disease by a factor of 2.48 (Kannarkat et al., 2015). In addition to pesticide exposure, water pollutants, heavy metal exposure, noise, rural living, and agricultural occupation, among many others, can affect the onset and development of PD (Ascherio and Schwarzschild, 2016; Collaborators, 2019). While MAPT-related PD is primarily linked to familial PD, environmental influences have less of an impact on its allelic pathogenesis (Hill-Burns et al., 2014). It’s like a puzzle where genes and environment pieces fit together to influence PD. Learning about these interactions helps us understand more about how PD happens and could even help in the future to figure out who might be at higher risk and how to prevent it.

## 8 Other practice

### 8.1 Calcium

It is noteworthy that specific groups of neurons, such as those found in the parietal region of the substantia nigra pars compacta, exhibit self-generated pacing and rely on L-type voltage-gated Ca2+ channels (Cav1.3) to facilitate the entry of calcium into the cell (Chan et al., 2007). This increased level of calcium entry is linked to heightened oxidative stress, mitochondrial impairment, and cellular demise (Vijiaratnam et al., 2021). The dihydropyridine channel blocker, isradipine, is sensitive to the central nervous system and blocks Cav1.3 or Cav1.2 L-type channels. It has demonstrated neuroprotecNtive properties in animal models exposed to dopamine toxin (Ilijic et al., 2011). Nonetheless, the results from a Phase III clinical trial were not as promising (Investigators, 2020).

### 8.2 Iron

One hallmark of PD is iron overload in the substantia nigra compacta area (Dexter et al., 1987). Because iron overload increases mitochondrial oxidative stress, which in turn causes α-synuclein to accumulate and aggregate and neuronal apoptosis, it can lead to neuronal loss (Vijiaratnam et al., 2021). Moreau et al. conducted a related experimental study using iron chelators that showed great potential (Moreau et al., 2018).

### 8.3 GLP-1

Type 2 diabetes is frequently treated with GLP-1 receptor agonists. Additionally, found in the brain are GLP-1 receptors, and agonists have demonstrated advantages in animal models of both dopaminergic and α-synuclein disorders. Possible explanations for these advantages might involve lower inflammatory responses (Chen et al., 2018) and α-synuclein buildup (Zhang et al., 2019). In an open-label Phase II research, exenatide was found to ameliorate the disease’s cognitive and motor symptoms (Nalls et al., 2019). Moreover, there are more trials underway to investigate the role of GLP-1 receptor agonists in alleviating symptoms of PD.

A growing number of clinical trials targeting specific genes for PD are underway. In addition to searching for therapeutic modalities targeting the causative factor, there is also confirmation of the clinical serendipity in discovering drugs that can alleviate the symptoms of PD through the genetic pathway in reverse. A study investigating the response phenotypes of Parkinson’s drugs, their gene target pathways, and pathological processes reinforces our belief that precision therapy for PD is possible in the future. Zonisamide was first created as an antiepileptic medication, however, it has now been discovered to significantly reduce PD symptoms. Recently, Tatsuhiko et al. discovered that the regulation of glutamate-associated synaptic activity and the p53 gene-mediated protection against the loss of dopaminergic neurons are primarily responsible for the beneficial effects of zonisamide on PD. Additionally, the immune system also plays a role in this process (Naito et al., 2022).

However, further investigation into prodromal and clinical data is necessary. The future remains challenging due to the limited availability of examination methods and the absence of established clinical models. In addition, we cannot ignore the variability caused by geography, which may lead to genetic mutations.

## 9 Conclusion and future directions

The extensive work of PD genome-wide association studies (PD GWAS) has identified an increasing number of loci associated with an increased risk of the disease. By integrating expression, epigenetic, and genomic association studies, candidate genes for PD are identified (Kia et al., 2021). Understanding the genes and mechanisms behind these loci is crucial for comprehending the pathogenesis of PD. Research on genetics, pathological progress, and symptomatology makes it possible to distinguish between healthy controls and patients via machine learning algorithms or multigene risk scores. Additionally, it may be applied to forecast different patient subgroups, age of onset, genotypes, and clinical patterns (Escott-Price et al., 2015; Nalls et al., 2015, 2019). In the age of machine learning and big datasets, there are a lot of prospects for PD diagnosis and therapy.

**Figure 4:**
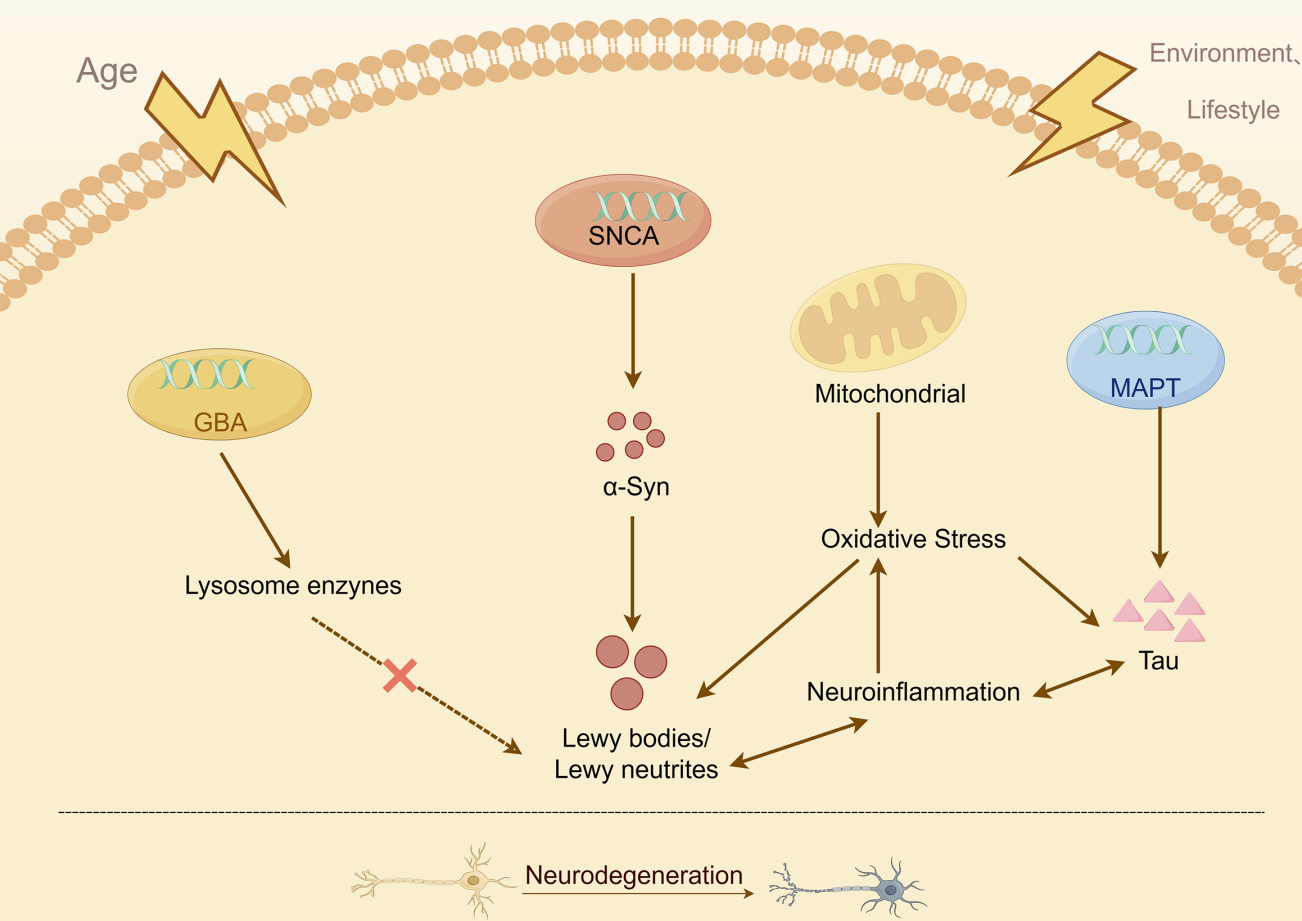
Simplified overview of the known mechanisms leading to neurodegeneration in PD.

Highlighting this, common mechanisms among subtypes have been noted for a long time, further demonstrating the important role of subtypes in the precise treatment of PD and the necessity of multiple pathways converging to trigger disease pathology. Pathological development, genetic progression, and subtyping of PD are important factors in identifying new biomarkers and therapies for the disease. The fact that reassuring therapy is not influenced by environment, lifestyle, ethnicity, or other variables is reassuring. Instead, it is individualized based on genes, pathogenic pathways, and clinical manifestations. In the future, PD may no longer be seen as a single pathological entity but rather as a condition that can be divided into subtypes with varying prognoses and responses to treatment. This division would allow for precision therapy (Berardelli et al., 2013; Figure 4). PD has a diverse phenotype and low heritability. While the underlying disease mechanisms are still being debated, cohorts of deep phenotypes have been developed to collect detailed, fine-grained data. These cohorts will help us study the underlying biological pathways and risk factors in order to identify therapeutic targets for advancing precision medicine (Schalkamp et al., 2022).

It must be recognized that research into precision Parkinson’s therapy, based on existing knowledge of genetics, is currently facing many dilemmas at both the clinical and preclinical stages. These dilemmas include the selection of preclinical models and the failure to recruit appropriate patient subtypes (Vijiaratnam et al., 2021). At the same time, there is insufficient research evidence to explain the interaction between multiple genes related to PD. To achieve this goal, the field should collect cells and tissues longitudinally from individuals with PD who are at genetic and environmental risk, not only those with clinical symptoms. This will allow for comprehensive genetic, transcriptional, and mechanistic analyses (Tansey et al., 2022). This will lead to a deeper understanding of the mechanisms underlying the pathogenesis of the disease, which will facilitate targeted drug research.

No gene, clinical feature, or disease pathway exists in isolation; each is interconnected and not specific to one condition. Disease stratification and typing will demonstrate their superiority in the future when dealing with complex and multifaceted clinical diseases (Chan, 2022). Research on novel genes that cause PD, combined with studies on deficits of α-synuclein, mitochondria, immune system, and lysosomes, will help identify new and overlapping mechanisms of dysfunction. This will enhance our understanding of the disease’s onset and progression. These insights from mechanistic studies and the resulting therapeutic opportunities may also have implications beyond PD.

## Author contributions

QW: Writing – original draft, Writing – review & editing. XG: Supervision, Validation, Writing – review & editing. LY: Validation, Visualization, Writing – review & editing. YJ: Supervision, Writing – review & editing. JZ: Software, Writing – review & editing. JH: Funding acquisition, Writing – review & editing, Writing – original draft.
